# mHealth and Digital Innovations as Catalysts for Transforming Mental Health Care in Ghana

**DOI:** 10.9745/GHSP-D-24-00062

**Published:** 2024-12-20

**Authors:** Enoch Sackey, Angela Ofori-Atta, Sammy Ohene, Kwadwo Obeng, Dror Ben-Zeev

**Affiliations:** a University of Washington, Seattle, WA, USA.; b University of Ghana, Legon, Ghana.; c Accra Psychiatric Hospital, Accra, Ghana.; d Korle Bu Teaching Hospital, Accra, Ghana.

## Abstract

How mHealth and digital innovations are key to transforming mental health care in Ghana, bridging gaps in a system challenged by resource scarcities and a critical shortage of mental health professionals.

## Key Messages

- Despite Ghana’s commitment to universal health coverage and the provision of mental health care in public institutions at no direct cost, the mental health system faces an overwhelming demand and logistical challenges, including a critical shortfall of mental health care professionals, geographic limitations, and stigma.
- This commentary explores the challenges and opportunities within Ghana’s mental health care system, emphasizing the critical role of mobile health (mHealth) and digital innovations in transforming mental health care delivery.
- We highlight the role of mHealth interventions, including initiatives like the M-Healer smartphone toolkit that equips traditional healers with mental health management techniques, and collaborations with digital communities, such as the West African Digital Mental Health Alliance, in improving mental health services and developing culturally relevant digital mental health solutions.
- Strategic actions that are essential for further advancing Ghana’s mHealth landscape include establishing dedicated training programs for clinicians to build capacity in digital technologies and integrating digital health solutions into hospital systems.

## INTRODUCTION

While Ghana is committed to universal health coverage, exemplified by the provision of mental health care in public institutions without direct costs, there remain challenges in translating this commitment into effective practice.^1^^,^^2^ The mental health system often grapples with an overwhelming demand and logistical hurdles, creating a discrepancy between the ideal of universal access and the actual delivery of mental health services.^3^^,^^4^

Ghana’s mental health care system struggles with significant shortages in clinical resources, infrastructure, and personnel, severely limiting its ability to meet the population’s mental health needs. The country faces a significant shortfall in mental health professionals, with only 1 psychiatrist for every 1.5 million people, far below the World Health Organization’s recommended ratio of 1 psychiatrist per 100,000 population.^5^ Clinical psychologists and psychiatric nurses are also in short supply, contributing to the fact that while an estimated 3.8 to 6.6 million Ghanaians live with moderate to severe mental health disorders, such as depression, anxiety, schizophrenia, and bipolar disorder, only about 2% receive formal treatment.^5^^,^^6^ Disparities in care access are particularly pronounced between urban and rural areas. The southern regions, where the country’s 3 specialized psychiatric hospitals are located, benefit from better access compared to rural and northern areas, leaving significant gaps in service delivery.

In addition to workforce shortages, stigma continues to be a major barrier, deterring many from seeking care. The availability of psychiatric medications is inconsistent, especially in rural areas, due to supply chain and infrastructure challenges. Although the Mental Health Act of 2012 aimed to address these issues by expanding services and increasing the number of community psychiatric nurses, underfunding and slow implementation have hindered progress.^5^ While the National Health Insurance Scheme provides partial coverage for mental health services, it remains insufficient to meet the country’s mental health needs.

The widening gap between mental health needs and available services underscores the need for innovative solutions that can bridge the divide and enhance the delivery and effectiveness of mental health care in the country. Mobile health (mHealth) technologies and other digital health innovations hold significant promise for enhancing mental health care in Ghana.

The widening gap between mental health needs and available services underscores the need for innovative solutions that can bridge the divide and enhance the delivery and effectiveness of mental health care in Ghana.

In this commentary, we explore some of the barriers that characterize mental health care delivery in Ghana. We examine factors, such as historical legacies, geographical constraints, and resource limitations, to provide a nuanced understanding of Ghana’s current mental health landscape. We also highlight how Ghana’s existing technological infrastructure can be harnessed to tackle these challenges.

## CONTEXT AND CHALLENGES OF GHANA’S MENTAL HEALTH CARE SYSTEM

In the early days of colonial rule in the 19th century, individuals with mental illnesses in Ghana (formerly Gold Coast) were predominantly confined to prisons.^7^ Those who were not incarcerated either managed on their own or sought help from traditional healers.^8^ However, with the introduction of the Lunatic Asylum Ordinance in 1888 by the colonial government, a structured approach to mental health care began to take shape. It wasn’t until 1904 that the Accra Psychiatric Hospital was established, marking the beginning of institutional psychiatric care in Ghana. In the years following independence in 1957, 2 additional facilities were founded: Ankaful Psychiatric Hospital near Cape Coast in 1965 and Pantang Psychiatric Hospital in 1975.^7^

These 3 specialized psychiatric hospitals, all situated in the more relatively developed southern region near the nation’s capital, where colonialists settled, formed the backbone of Ghana’s psychiatric care infrastructure. Their southern concentration presented significant access challenges for many, particularly those residing in the northern and more remote areas.^5^ However, since the Ghana Mental Health Act was passed in 2012, there have been significant improvements in making mental health services more accessible. These include establishing psychiatric units in all 5 teaching hospitals across 5 regional capitals, 2 of which already have psychiatric hospitals. Additionally, every district hospital now features a mental health unit staffed with at least 1 specialized nurse.^9^

These additional psychiatric units and district hospitals have made mental health services relatively more accessible to a wider population. However, there are still pressing challenges, including significant shortage of staff and limited resources. Furthermore, for those living outside the regional and district capitals, accessing these facilities involves long, exhausting journeys. This not only disrupts ongoing treatment but also places additional burdens on patients and their families. Consequently, many turn to readily accessible but less regulated alternatives like faith-based healing centers, which often serve as the first point of call within their communities.^5^

In Ghana, approximately 4.5% of the gross national product is allocated to health care, with a modest 1.3% specifically designated for mental health services and facility maintenance.^10^ Despite these budget constraints, it’s important to note that not all mental health facilities are in decline. However, in some areas, the existing infrastructure still falls short of modern standards. The physical aging of mental health facilities, as noted,^11^ can unintentionally reinforce societal stigma around mental health services and those who seek them. Aging infrastructure often signals a lack of investment and modernization, leading to perceptions that mental health services are inadequate or neglected. This can deepen existing stigma not only around psychopathology but also around the type of person who seeks care at such facilities. People may hesitate to access these services for fear of being seen and associated with outdated, underfunded institutions, reinforcing stereotypes of individuals relying on public aid or connotations with welfare and poverty.

This stigma, reinforced by the condition of mental health care facilities, can discourage individuals from seeking the care they need.^12^ This is further complicated by geographical barriers, making the mental health care system seem fragmented and less accessible. The term “fragmented” is used to describe the way the mental health care system in Ghana is divided and unevenly distributed, resulting in inconsistent and often inadequate service provision, particularly for those in rural and remote communities. In these areas, where the influence of traditional beliefs is stronger, limited access to care often coincides with belief systems that view mental disorders as having supernatural origins.^13^ This cultural viewpoint drives many to opt for traditional or spiritual-based healing that uses practices, such as shackling, flogging, and forced fasting, that can be traumatizing and potentially worsen mental health conditions.^13^

## GHANA’S TELECOMMUNICATION INFRASTRUCTURE AND READINESS FOR MHEALTH

Amid these challenges, mHealth and digital innovations present a viable solution. mHealth refers to the use of mobile devices, such as smartphones and tablets, to support medical and public health practices. This includes the use of mobile apps, text messaging, and other mobile technology to deliver health services and information. Digital health encompasses a broader range of technologies beyond mobile devices. It includes telehealth, electronic health records, health information systems, wearable devices, and other digital tools that enhance the delivery and management of health care services. As highlighted in the literature,^14^ these technological interventions are not intended to replace health care providers but to augment their ability to bridge gaps in care access. They are particularly effective in offering remote access to mental health services, circumventing issues related to inadequate infrastructure and geographical disparities.^15^ They offer a discreet and accessible option for those reluctant to seek help due to stigma. mHealth is particularly useful for remote consultations, health monitoring, and the dissemination of health-related information, making it a powerful tool for improving access to health care services in underserved areas. Digital health aims to improve health care outcomes by leveraging technology to increase efficiency, reduce costs, and provide more personalized care. This approach can mitigate the shortage of psychiatrists and psychologists by using digital platforms to connect patients with mental health professionals. Digital tools can facilitate continuous monitoring and follow-up for patients with chronic mental health conditions. This ensures that patients receive consistent care and support, reducing the likelihood of relapse and improving overall treatment outcomes. In general, mHealth and digital innovations present critical options for culturally adapting interventions and expanding accessibility and reach.^14^^,^^16^

Ghana showcases a promising landscape for the integration of telemedicine and technology into mental health care delivery. The country’s readiness for this transformative shift becomes evident when considering the convergence of various favorable elements and advancements, notably the burgeoning telecommunication infrastructure and the government’s steadfast support for technological development.^17^ The remarkable growth of telecommunications infrastructure in Ghana has transformed the country’s digital landscape, making mobile phone usage nearly ubiquitous throughout the nation. This widespread adoption of mobile technology cuts across various sectors and demographics.^18^^,^^19^

In Ghana, mobile phones come in various types, ranging from basic feature phones to advanced smartphones with sophisticated computational capacities, supported by a robust 4G network that extends even to remote areas. Approximately 67% of the population are unique mobile subscribers, with mobile Internet penetration at about 45%. The majority of mobile users are on prepaid plans, which offer flexibility and affordability, making them accessible to a broad segment of the population. Prepaid subscriptions account for more than 90% of the mobile market, reflecting the economic realities and preferences of Ghanaians.^20^ The flexibility of prepaid plans allows users to manage their data usage according to their needs and financial capacity, making mHealth apps accessible to a larger population. The existence of this technological infrastructure, coupled with the government’s commitment to enhancing information and communication technology literacy, paints a promising future where mHealth can assume a pivotal role in the delivery of mental health care services.

The existence of Ghana’s technological infrastructure paints a promising future where mHealth can assume a pivotal role in the delivery of mental health care services.

The readiness and acceptance by Ghanaians for mHealth innovations are further supported by a study examining Ghanaians’ readiness for mHealth^20^ and collaborative initiatives like the West African Digital Mental Health Alliance (WADMA).^17^ The Technology Acceptance Model,^21^ a widely recognized framework, provides valuable insights into the Ghanaian context. According to the Technology Acceptance Model, an individual’s willingness to embrace mHealth services hinges on their belief in the technology’s effectiveness and their own ability to use it.^20^ In the case of Ghana, the evidence of high level of acceptance and readiness among the population, as indicated in the literature,^20^ not only signals cultural openness to technological innovations but also indicates an acknowledgment of their practicality and feasibility.

WADMA exemplifies a proactive approach to the development and adoption of digital mental health solutions. This mission-driven network fosters collaboration between technologists and clinical researchers, originating from partnerships between institutions in the United States and West Africa, including the University of Washington, the University of Ghana, and the University of Ibadan.^17^ WADMA’s goal is to nurture a new generation of digital mental health researchers and practitioners dedicated to advancing mental health services through digital innovations and cultural adaptations of care.

Such innovations align with initiatives like the Mobile Doctors Network, a public-private partnership offering free mobile-to-mobile voice and text messages to doctors registered with the Ghana Medical Association.^22^ The Mobile Doctors Network, in operation since 2004, demonstrates the benefits of a cost-effective communication network for physicians, enhancing clinical consultations and patient referrals.^23^ Similarly, Mobile Technology for Community Health, a World Bank project, empowers health workers to digitize service delivery information and monitor patient care.^24^ The project’s use of mobile technology has been instrumental in improving access to maternal and child health information and services, leading to timely health interventions that have a positive impact on maternal and child health outcomes.^24^

As individuals who are affiliated with major psychiatric hospitals in Ghana, we believe that Ghana’s embrace of mHealth interventions, especially during the COVID-19 pandemic, highlights the significant impact of technology in health care. For example, throughout the pandemic, psychiatric hospitals in Ghana effectively employed audio and video consultations, albeit on a limited scale, to maintain continuity in mental health services.

More recently, the M-Healer smartphone toolkit has been created to help improve exposure to evidence-based psychosocial interventions and education focused on preservation of human dignity and safety in clinical practice.^14^^,^^16^ By equipping traditional healers with educational materials and tutorials on essential mental health management techniques, such as de-escalation, deep breathing, and reframing anxious thoughts, M-Healer bridges the gap between traditional healing practices and contemporary, evidence-based mental health care. This integration aims to support paraprofessionals’ delivery of higher-quality care while reducing their use of practices that constitute human rights violations.^14^ M-Healer is specifically designed for users in Ghana, and it is unique in that it provides content in both Ghanaian language, Twi, and English, increasing cultural relevance and accessibility among lay practitioners. Furthermore, the app has been carefully designed to address the challenges posed by limited Internet connectivity and high data costs. Its offline functionality, featuring optimized digitally animated videos and audio files, enables use without the need for an active Internet connection. This offline capability, coupled with a user-friendly dark mode interface, enhances the app’s practicality by ensuring efficient battery consumption, all without necessitating complex backend functionality, active programming interface integration, or database connections.^16^

The M-Healer smartphone toolkit bridges the gap between traditional healing practices and contemporary, evidence-based mental health care.

The M-Healer app has demonstrated significant acceptability and promising real-world use in Ghana. In a user trial, healers who received smartphones with the M-Healer app engaged with it frequently, averaging nearly daily use, and self-initiated app interactions were higher than expected.^25^ Patients saw notable improvements in psychiatric symptoms, psychological distress, and stigma. Additionally, there was a significant reduction in human rights abuses, such as chaining, suggesting that M-Healer may influence positive behavioral changes among healers. The intervention, combining mHealth technology with pharmacotherapy provided by a visiting nurse, proved feasible, safe, and clinically effective, further solidifying its potential as a scalable tool for improving mental health outcomes in Ghana.^25^

Over the past decade, Africa has seen a surge in mHealth initiatives aimed at addressing health care challenges, leading to numerous pilot projects, especially those using short message service-based interventions. This period highlighted the potential and limitations of mHealth technologies and introduced the phenomenon of “pilotitis,”^26^ where small-scale pilots often failed to scale to national programs, frustrating stakeholders. Despite these challenges, notable successes include Uganda’s mTrac System, which scaled nationally within a year, significantly improving data timeliness, reducing medicine stock-outs, and facilitating real-time health monitoring. Similarly, China’s IDRIMS, developed in response to the SARS outbreak, scaled rapidly due to strong political will and substantial investment.^26^ To avoid “pilotitis” in Ghana and ensure mHealth initiatives have a national impact, strategies such as integration with existing health systems, securing long-term funding, engaging local communities, and developing training programs for health care providers are essential. The M-Healer toolkit, for example, has been successfully integrated into the larger WADMA program, showcasing the potential for mHealth to strengthen the mental health care system and achieve sustainable, nationwide improvements.

## SUGGESTIONS TO ACCELERATE MOBILE HEALTH IN GHANA

Overall, Ghana’s mHealth landscape is rapidly evolving. To further accelerate this evolution, several strategic policies and actions are essential.

First, the establishment of dedicated training programs for both practicing clinicians and those in training is crucial. This will include building a workforce skilled in digital health technologies. Incorporating this specialized training into existing medical and nursing curricula, coupled with offering certifications or recognition upon completion, would likely boost participation and enhance proficiency in digital health practices.

Second, the integration of digital health solutions into hospital systems is crucial for streamlining processes and enhancing patient care. The Ghanaian Ministry of Health has already made significant progress in this area, with policies encouraging, as well as mandating, the adoption of electronic health records and telemedicine consultations. This policy is currently at an advanced stage of implementation. Continued support for this initiative, including funding for infrastructure upgrades and technical support, will be essential for its success.

Finally, collaboration with initiatives like WADMA can be instrumental in developing and adopting culturally relevant digital mental health solutions. Such partnerships can focus on research, funding, and implementation support, helping to tailor digital mental health interventions that resonate with the unique needs of Ghana’s population.

By aligning these strategic actions with Ghana’s commitment to universal health coverage, the nation can enhance its position as a leader in digital health innovation in West Africa. The transformation of Ghana’s mental health care system is a necessity; through creative integration and development of mHealth and other democratizing digital mental health technologies, the promise of more accessible mental health care can become a reality.

## Notes

**Peer Reviewed**

**First Published Online:** November 5, 2024.
